# The morpho-genetic and ecological niche analyses reveal the existence of climatically restricted *Cycas zeylanica* complex in Sri Lanka

**DOI:** 10.1038/s41598-019-53011-w

**Published:** 2019-11-14

**Authors:** Asanka Mudannayake, Lahiru Ranaweera, Preminda Samaraweera, Suneth Sooriyapathirana, Anoma Perera

**Affiliations:** 1 Department of Botany, Faculty of Science, University of Peradeniya, Peradeniya, Sri Lanka; 2 Postgraduate Institute of Science, University of Peradeniya, Peradeniya, Sri Lanka; 3 Department of Molecular Biology and Biotechnology, Faculty of Science, University of Peradeniya, Peradeniya, Sri Lanka

**Keywords:** Biodiversity, Ecological modelling

## Abstract

Taxonomy and phylogenesis of Sri Lankan cycad species of the subsection *Rumphiae* has not been fully resolved and therefore, we conducted an island-wide survey of cycads of the subsection to assess their morphological or genetic variations while exploring the phylogenetic relationship between Sri Lankan *Rumphiae* and other world cycad species. Further, we assessed the possible distribution of the species in the region through climatic profiling, using maximum entropy modeling approach. We analyzed 21 variable morphological features in collected specimens and used the polymorphism of *trnH-psbA* locus to understand the phylogeny. The distance tree drawn from the principal component analysis revealed a significant variation in female reproductive structures. The maximum likelihood tree separated Sri Lankan *Cycas zeylanica* to a well-supported unigeneric clade (bootstrap = 96, posterior probability = 100) with shallow divergence. Ecological niche modeling supported the existence of *Cycas zeylanica* in South East Asia and in southern Western Ghats in India in addition to the Wet Zone of Sri Lanka. We rename the taxa as *Cycas zeylanica* complex based on the observed high morphological diversity of female reproductive structures which might have ascended due to multiple introductions of South East Asian cycads by long distance dispersal of seeds through sea currents.

## Introduction

The genus *Cycas* which belongs to the unigeneric family Cycadaceae, is considered as the basal lineage of the order Cycadales^1^. To date, 115 species are reported under the genus *Cycas* worldwide^2^, and of these, around 40 species occur in the Indo-Chinese region^3^. Inherent characters of cycads such as slow-growth, a heavy natural variability of morphological traits^4^ and dioecious nature made the identification of individuals to the species level difficult in some instances, but identifying to the section or subsection levels merely based on morphological features is not challenging. Thus, six sections of the genus *Cycas* have been recognized and often used in taxonomic descriptions. Four of these sections *viz*., *Cycas*, *Asiorientales*, *Indosinenses* and *Stangerioides*^5^ had been identified initially, but later two other sections, i.e*. Wadeae*^6^ and *Panzhihuaenses*^7^ have been added to the classification system. Of these, the section *Cycas* is further divided into three subsections: *Cycas*^8,9^, *Endemicae*^9,10^ and *Rumphiae*^8,9^.

The combined feature of the presence of spongy tissues in the endotesta or the presence of a fibrous layer in sarcotesta of the seed is a primary attribute of taxonomic significance in differentiating the individuals of the three subsections of the section *Cycas*^8,11^. Thus, this feature allows the differentiation of Sri Lankan cycads into two subsections, *Cycas* and *Rumphiae*^12,13^. Owing to the fact that there are many similarities in morphological features among different Sri Lankan taxa, identifying these to the species level is practically difficult^13^. Reproductive morphological characteristics of individuals of the subsection *Rumphiae* in Sri Lanka vary dramatically though these show a restricted distribution within the country, reflecting complex evolutionary relationships among Asian cycads.

Presence of *C. rumphii* Miq. (subsection *Rumphiae*) in Sri Lanka has initially been recorded in the *Revised Handbook to the Flora of Ceylon*^14^, but later, the occurrence of *Cycas zeylanica* (J. Schuster) A. Lindstrom and K. D. Hill in Sri Lanka was confirmed by Lindstrom and Hill^12^. However, there are no records available to negate the presence of *C. rumphii* in Sri Lanka. Therefore, by an island-wide survey of individuals of the genus *Cycas* in Sri Lanka, Mudannayake *et al*.^13^ have studied 26 reproductive and 23 vegetative features of individuals of the subsection *Rumphiae* and observed the presence of substantial continuous variation in many of the important morphological features among the individuals, which led to describe the group as *C. rumphii* complex. In general, the features of the megasporophyll which is crucial in delimiting the subsection *Rumphiae* species^8,13,15,16^ show a considerable variation. The existence of such a substantial variation of morphological features within the subsection *Rumphiae* in Sri Lanka could be attributed to many factors including their ecological niches or dispersal pathways, and need to be further investigated.

The spongy tissues present in seeds of the subsection *Rumphiae* plants make them buoyant in seawater, allowing their seeds to disperse over different localities. As many researchers have explained^17–19^, seed dispersal through seawater may lead to the successful recolonization of plants in new regions and to form genetically distinct forms in colonized locations. As such, hybridization among the members of the subsection *Rumphiae* could have occurred in introduced locations^10,20^. Due to the genetic mixing caused by hybridization, the taxonomy of the subsection *Rumphiae* based on morphological features has become very complicated, leaving molecular characterization as the sole means of identification and characterization of species. Thus, it is vital to revisit the subsection *Rumphiae* in Sri Lanka along with other cycads using molecular systematics.

Molecular phylogenetics is a reliable tool in taxonomic and phylogeographic studies. Nucleotide polymorphism of conserved gene regions is a key component in explaining the genealogies. In plants, most of the taxonomically essential gene regions are found in the chloroplast. The *trnH-psbA* locus, a non-coding intergenic spacer region in the plastid genome, is considered as the most appropriate single locus barcoding region for plants^21^ due to the presence of both SNP (Single nucleotide polymorphism) and INDELs (Insertions and deletions)^22,23^. Moreover, *trnH-psbA* exhibits higher mean percent sequence divergence at generic level enabling a good resolution power^21^. The application of *trnH-psbA* sequence polymorphism to identify species is a routine practice in plant systematics^23^. The *trnH-psbA* has become a popular barcoding locus for Cycadaceae as well^24–26^. The length of *trnH-psbA* sequences in Gymnosperms ranges from 283–1006 bp^23^, and with good priming sites, short read lengths (~ 600 bp) can be efficiently sequenced across many taxa.

In the present study, we aimed to reveal the presence of morphological or genetic variations within the subsection *Rumphiae* in Sri Lanka, explore the phylogenetic relationship between Sri Lankan *Cycas zeylanica* and other world cycads, and explain the possible distribution of the species using climatic profiling through Ecological Niche Modeling (ENM) analysis.

## Materials and Methods

### Sampling

**Figure 1:**
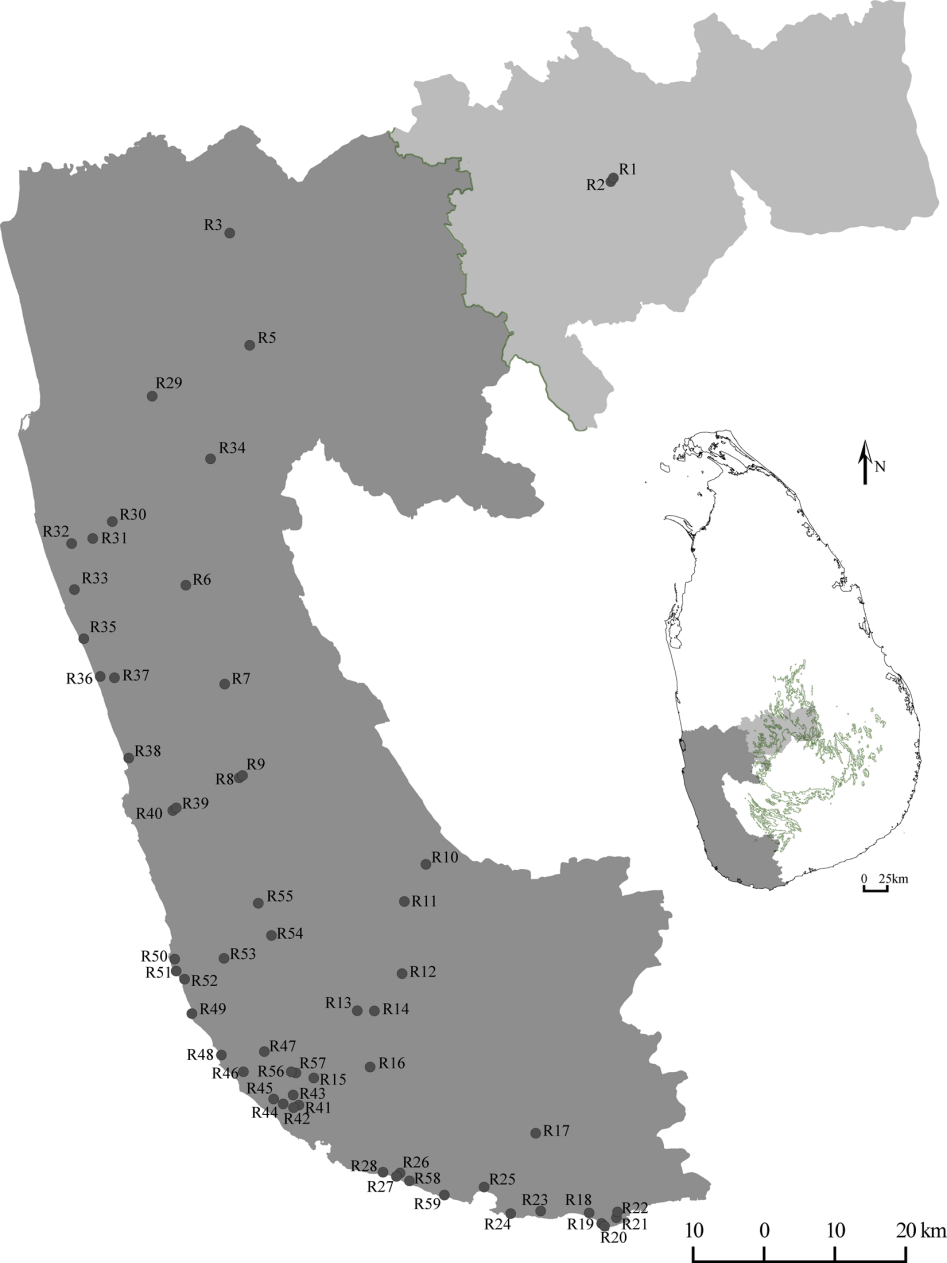
The locations of the individuals examined, of subsection *Rumphia*e in Sri Lanka. The contour at 1,067 m (3,500 ft) is shown in green lines in the map of Sri Lanka.

We conducted an island-wide survey by examining about 200 individuals of Sri Lankan cycads using the morphological features explained by Mudannayake *et al*.^13^. We considered a total of 49 morphological features (Supplementary Table S1) and recorded only the features available at the time of sampling. We identified that 53 individuals belong to the subsection *Rumphiae* and all of these except two cultivated plants (R1 and R2) inhabit the lowland Wet Zone of Sri Lanka (Fig. 1) where the elevation is below 1,067 m (3,500 ft). Also, we noted that the frequency of occurrence of individuals is comparatively higher near the coast than inland. Evidence proves that the above mentioned two individuals sampled in the highland Wet Zone are cultivated plants and have not occurred due to natural regeneration. We photographed and made line-diagrams of female and male reproductive structures available in the individuals assessed.

### Morphological data analysis

Out of the 53 individuals found, we examined morphological (vegetative, female and male reproductive) features of 40 individuals which had attained the reproductive maturity. Out of these 40 individuals, female reproductive structures were available only in 25 individuals and male reproductive structures were available only in one individual. We did not find reproductive structures in the rest of the individuals. Therefore, we only considered 25 female plants with observable reproductive structures for the morphological data analysis. After initial examination of 49 morphological features (Supplementary Table S1), we removed less variable and monomorphic characters of the individuals studied, and thus, 21 quantitative and qualitative morphological features (11 vegetative and 10 female reproductive) were considered for the detailed analysis (Supplementary Table S1). We analyzed the data by variable reduction multivariate techniques^27^, Pearson’s correlation analysis for quantitative data, Spearman rank correlation analysis for qualitative data and Principal Component Analysis (PCA) for both qualitative and quantitative data by PCAmix function in PCAmixdata Package^28^ using R Statistical Software^29^. The presence of different groups was revealed by the morphological analysis based on a similarity matrix developed for the Principal Components (PCs) by dendrogram generated by Ward linkage method based on Euclidean distances.

### DNA extraction and sequencing

We extracted genomic DNA from the immature leaf samples of 17 individuals of the subsection *Rumphiae* representing different localities using DNeasy plant mini kit (Qiagen, Hilden, Germany). Polymerase Chain Reaction (PCR) was carried out in 40 µl reactions using 2 × GoTaq Green Master Mix (Promega Co, Madison, WI, USA) according to manufacturer’s protocol using the primers *psbA-trnH* (P_F_: 5′GTT ATG CAT GAA CGT AAT GCTC 3′^30^; P_R_: 5′CGC GCA TGG TGG ATT CAC AA TCC 3′^31^). PCR was carried out under the following conditions: 5 min denaturation at 94 °C, followed by 30 cycles of 94 °C for 1 min, primer annealing at 55 °C for 1 min and synthesis at 72 °C for 1 min and 30 s, with a final extension at 72 °C for 7 min^21^. We purified PCR products and subjected to the 3x cycle sequencing using a 3100 sequencer (Applied Biosystems).

### Phylogenetic analysis

We retrieved the sequence data of 30 individuals (in addition to the 17 sequences generated in the present study) for the locus *trnH-psbA* from GenBank to represent the total variation of the six subsections in the genus *Cycas* (Supplementary Table S2). To align 47 sequences, we implemented CLUSTAL W algorithm^32^ in MEGA V7^33^. To select the precise evolutionary model to fit the data, we implemented jModelTest V.2^34^ in CIPRES supercomputer^35^. We avoided oversimplification of the model selection by assessing across 88 models using more than 95 parameters. To choose the best model in Akaike Information Criteria (AIC)^36^, we used AIC weights and negative log-likelihood values. We carried out a tree search in RAxML^37^ using the rapid + boostrap algorithm for 1000 iterations in CIPRES science gateway^35^. The best scoring Maximum Likelihood (ML) tree was selected using log Likelihood values. Then all the bootstrap (bs) trees generated were implemented to produce one majority rule consensus tree. We used the best ML tree topology as the primary tree structure to interpret the bs values given in the majority rule consensus tree. To support our analysis further, we ran a Bayesian tree search using MrBayes 3.1^38^ in CIPRES Science gateway^35^. We implemented four Markov Chain Monte Carlo (MCMC) chains (two hot and two cold chains) for 50 million generations. The MCMC chains sampled trees in every 5000 cycles, and the analysis was set to discard the first 10% of the trees as burn-in. We used substitution parameter, the proportion of invariable sites and gamma shape parameters given by the evolutionary model to run this analysis. We assessed the Effective Sample Size (ESS) of the target distribution to measure the chain convergence to avoid the poor mixing and for the enhancement of the independent sampling of the sampled trees. The trees probed after maximum chain convergence were used to draw the 50% majority rule consensus tree. We used FigTree v1.4.3^39^ to edit the trees further.

### Ecological Niche Modeling (ENM)

We used 53 ‘presence-locations’ (i.e., total sampling effort) to model the predicted distribution of *C. rumphii* complex. We used geographic extant including most of the regions of South Asia and South East Asia where closely related cycads are known to occur. As the environmental covariates, we downloaded 19 bioclimatic variables (Supplementary Table S3) in Global Climate Databases^40^. Using ArcGIS Version 10.4^41^, we clipped 19 climatic layers into chosen geography. We used Maxent version 3.3.3k^42^ to carry out maximum entropy modeling. Here we did not use a Bias file as we are modeling the species distribution in full extent of selected geography, thus over fitting of the model could be avoided. The model fitting was carried out by using the Threshold Independent Method^43^ where the different β regulation parameters were changed (β = 0.1, 1, 2, 3, 4, 5) while assessing the Area Under Curve (AUC) parameters. We kept most of the other parameters default, as the Maxent chooses the best parameters for the analysis^44–46^. We ran the analysis for 5000 iterations using 1000 background points to increase the robustness of the model. We created the predicted distribution map using 10% threshold^47–49^.

## Results

### Morphology

Out of the 49 morphological features considered (23 vegetative, 20 female reproductive and six male reproductive traits) (Supplementary Table S1), 21 were found to be polymorphic, two were polymorphic but depend on the position of attachment on the tree, 17 were monomorphic, three were less variable, while six were not considered due to data deficiency. We considered only the 21 polymorphic traits of the female plants for the analysis which consists of 17 quantitative and four qualitative traits.

### Variability of the vegetative traits

**Figure 2:**
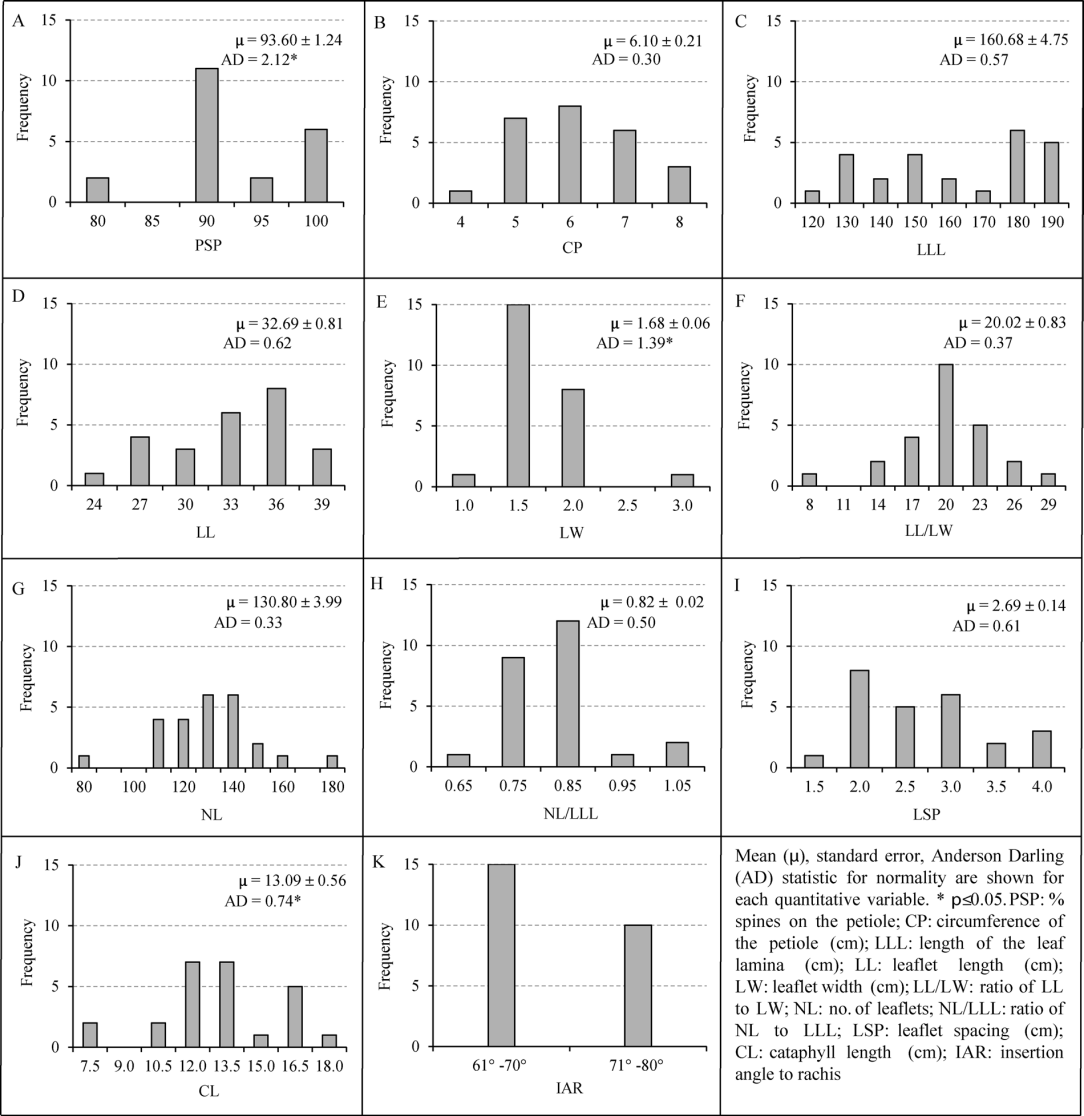
Variability of the vegetative features of the subsection *Rumphiae* in Sri Lanka. (**A**–**J**) quantitative traits, (**K**) qualitative trait.

The three vegetative traits, % spines on the petiole (PSP), leaflet width (LW) and cataphyll length (CL) (Supplementary Table S1) showed non-normal distributions as revealed by the significant Anderson Darling (AD) coefficient [(p ≤ 0.05) (Fig. 2A,E,J)] whereas the features circumference of the petiole (CP), length of the leaf lamina (LLL), leaflet length (LL), ratio of leaflet length to leaflet width (LL/LW), number of leaflets (NL), ratio of NL to LLL (NL/LLL) and leaflet spacing (LSP) were normally distributed [(p > 0.05) (Fig. 2B–D,F–I)]. The PSP of 90 was more common in the plants studied and fell within the mid-point of the range observed (Fig. 2A). The variation of the CP displayed quantitative and normal distribution with AD of 0.30. The parameters LLL, LL, LL/LW, NL, LSP and CL, displayed continuous quantitative distributions with very high variability. The only selected variable morphological feature of insertion angle to rachis (IAR) showed strict dimorphism with 3:2 observed ratio (Fig. 2K) and fitting into the dihybrid epistatic ratio of 9:7 (complementary gene action) (χ^2^ = 0.143, p = 0.0705).

### Variability of the female reproductive traits

**Figure 3:**
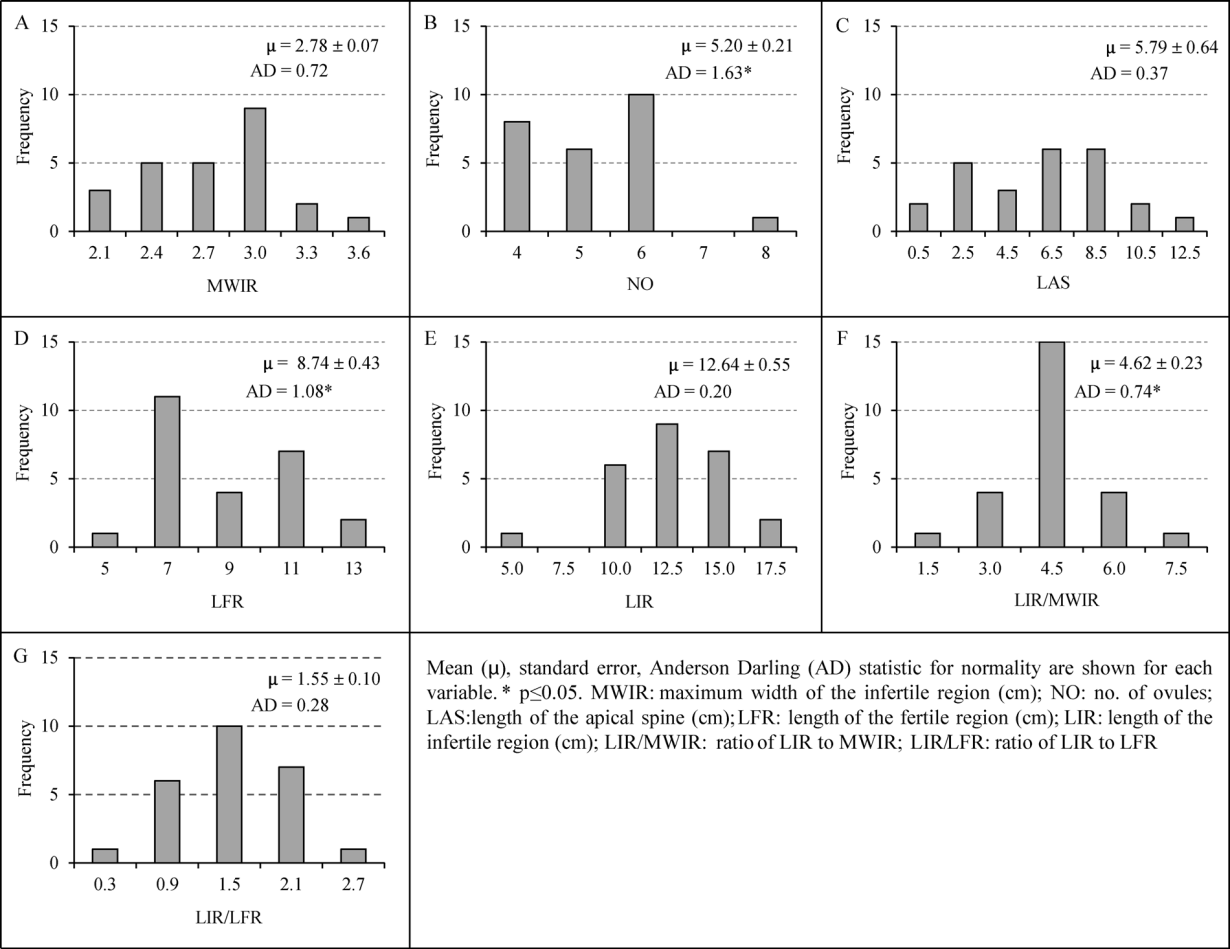
Variability of the quantitative traits of the female reproductive features of the subsection *Rumphiae* in Sri Lanka.

**Figure 4:**
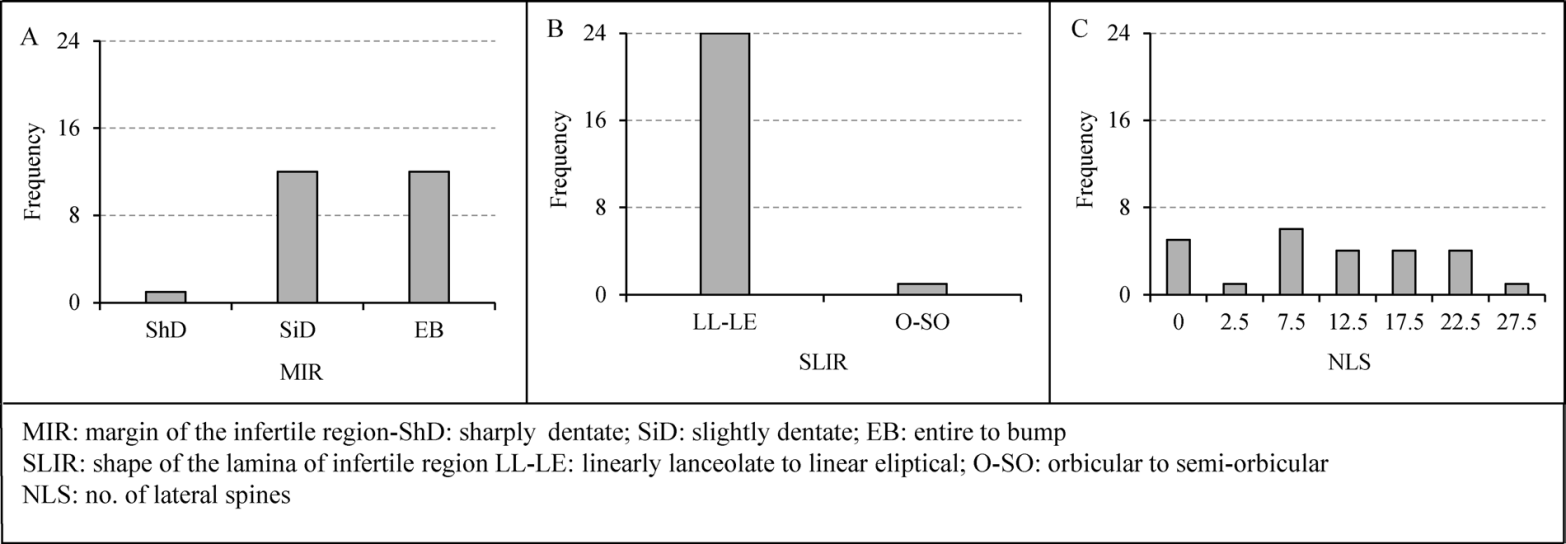
Variability of the qualitative traits of the female reproductive features of the subsection *Rumphiae* in Sri Lanka.

Except number of ovules (NO) (Fig. 3B), length of the fertile region (LFR) (Fig. 3D) and, ratio of length of the infertile region (LIR) to maximum width of the infertile region (LIR/MWIR) (Fig. 3F), the other quantitative traits exhibited normal distributions (Fig. 3A,C,E and G). The feature MWIR and LAS (Fig. 2A,C) showed higher variation across the individuals assessed. Out of the three quantitative polymorphic variables, the margin of the infertile region (MIR) showed trimorphic distribution (9:6:1, χ^2^ = 1.24, p = 0.538) (Fig. 4A) whereas shape of the lamina of the infertile region (SLIR) displayed dimorphic distribution (15:1, χ^2^ = 0.216, p = 0.642) (Fig. 4B).

### Correlation analyses

To ascertain the correlation among the quantitative vegetative and reproductive features the detailed Pearson Correlation Coefficient (PCC) values with the associated probability levels were calculated, and are given in the Supplementary Tables S4 and S5. The number of leaflets (NL) and the length of the leaf lamina (LLL) were strongly and significantly correlated (PCC = 0.71). However, CL and LLL were negatively correlated, and the strength of correlation was less (PCC = −0.42). The length of the apical spine (LAS) and LFR were correlated with NO (PCC = 0.59 and PCC = 0.53 respectively). A solitary significant correlation was observed between the qualitative traits MIR and NLS (PCC = −0.67).

### Cluster analysis based on the morphological diversity

**Figure 5:**
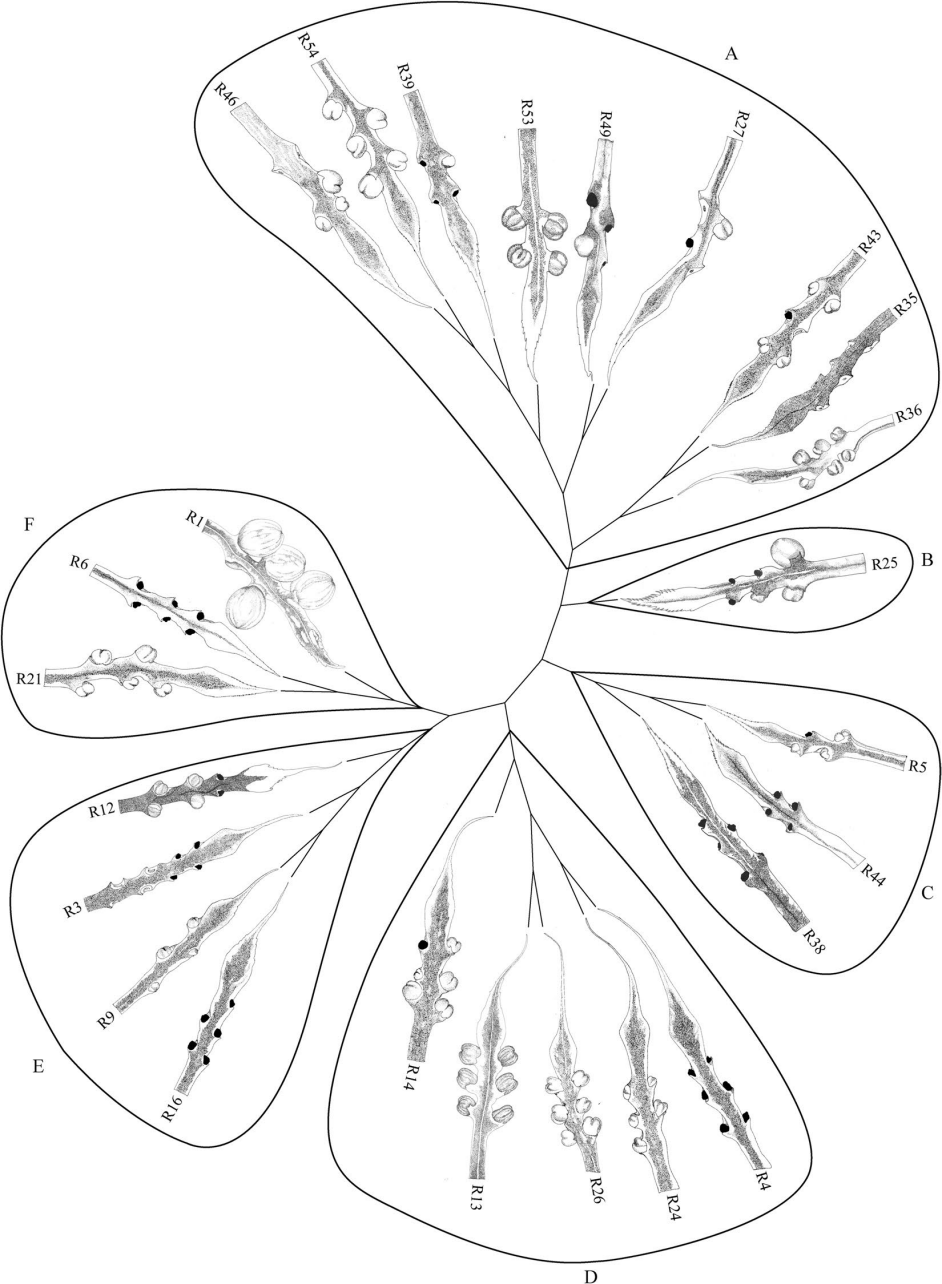
Dendrogram generated by Ward linkage method based on Euclidean distance drawn for all the Principal Components. The similarity among the samples was studied based on morphological traits and six distinct Clusters (**A–F**) were identified at 70% similarity. The megasporophyll structures of each individual of the subsection *Rumphiae* of Sri Lanka are given at the Operational Taxonomic Units of the dendrogram.

The selected polymorphic morphological features subjected to PCA, detected 12 PCs, which explained 91% of the total variability (Supplementary Table S6). The calculated PCs, explained variance and Eigen values are given in Supplementary Table S6. Based on all the PCs, we drew a distance tree and obtained six clear clusters at a morphological divergence of 30% (Fig. 5). The Cluster A included nine individuals, and the Cluster B included only one individual. The Clusters C and F contained three individuals in each and Clusters D and E contained five and four individuals respectively. The occurrence of extreme heterozygosity through hybridization is apparent from Fig. 5 as no two individuals were identical to each other.

### Phylogenetic analysis

**Figure 6:**
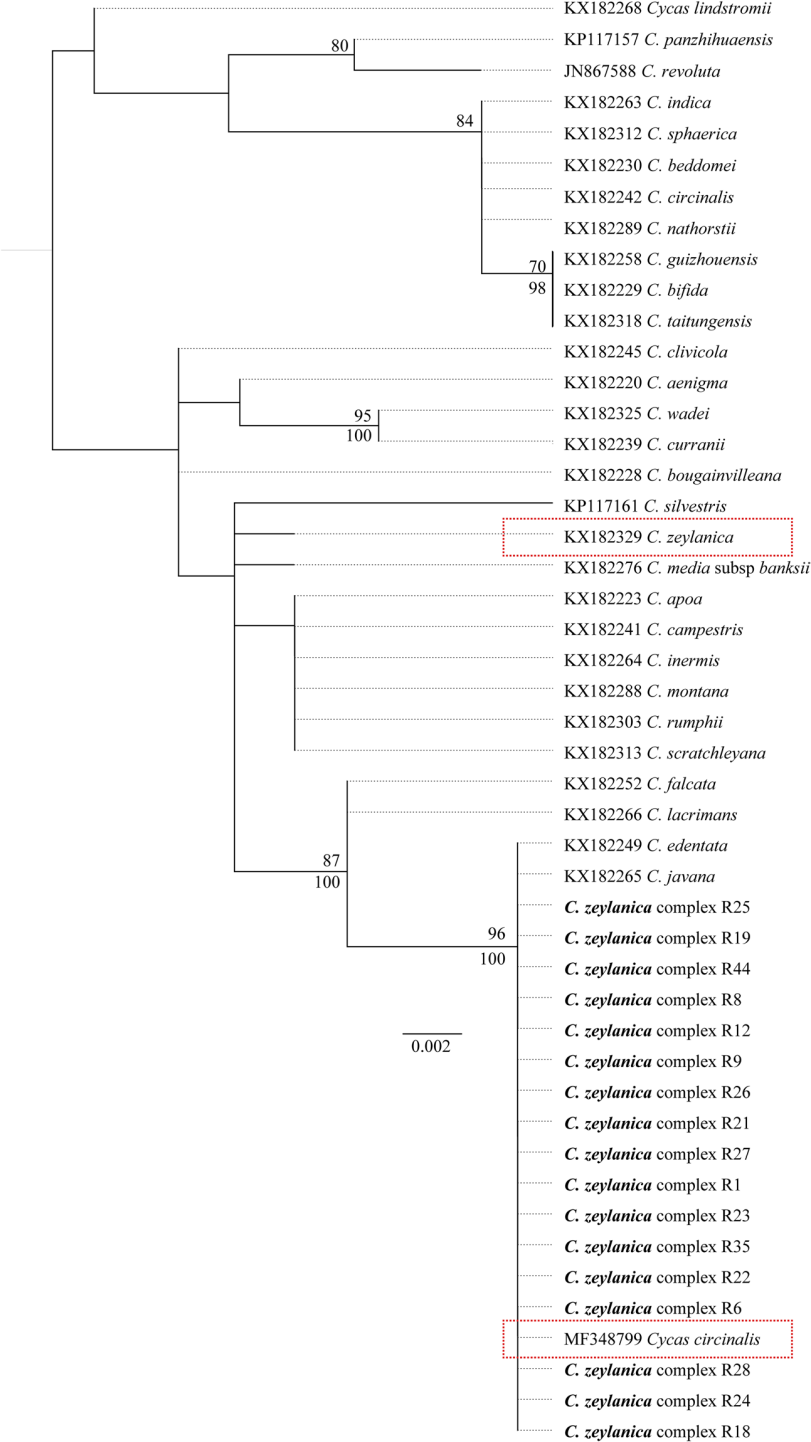
The best scoring Maximum Likelihood tree constructed in RAxML. The bs values are given above the node and PP values are indicated below the node. The bs values less than 70 and PP values less than 90 are not shown. Possibly misidentified individuals are highlighted in red boxes.

The sequenced gene region consisted of 611 base pairs. Of these 31 bases were polymorphic and only 16 sites were parsimony-informative while 14 sites were singleton. The details of the sequence polymorphism of *trnH psbA* are given in Supplementary Table S7. The TPMuf + I sub model was selected as the best evolutionary model to describe the dataset, during the model selection (negative log likelihood = 1063.24, AIC weight = 0.076). We used rate parameters (AC = 0.302, AG = 1.017, AT = −1.000, CG = 0.302, CT = 1.017 and GT = 1.000), proportion of invariable sites (0.66) and nucleotide frequencies [*f*(A) = 0.27, *f*(C) = 0.18, *f*(G) = 0.21 and *f*(T) = 0.34] of the evolutionary model to carry out the further analysis. Our tree search in Maximum Likelihood (ML) framework resulted in best scoring ML tree at a negative log likelihood value of 1062.74. The bootstrap values are indicated on the best scoring ML tree (Fig. 6). The MCMC chains converged maximum in the first 5000 runs which were discarded as burn-in. The ESS for sampled priors were above 200 indicating independent sampling from posterior distribution and reliable tree topology. The best scoring ML tree and the 50% majority rule consensus tree built in Bayesian framework had almost similar branching patterns. For the ease of representation, we only show the ML tree with imprinted node support values (Fig. 6). The Sri Lankan *Cycas Rumphii* complex (*Cycas zeylanica* complex *nov*.) formed a well-supported unigeneric clade (bootstrap = 96, posterior probability = 100) with almost identical individuals. However, the accession MF348799^26^ was also clustered within this clade, but its specimen has been identified as *C. circinalis* of the subsection *Cycas* and it is very unlikely to have members of subsection *Cycas* within this clade. It is possible that this could be a misidentification. The GenBank accession of *C. zeylanica* (KX182329) was also diverged out from the *C. zeylanica* complex clade *nov*., and it could also be a misidentification and must be revisited (Fig. 6). We submitted the sequences generated in the present study in GenBank under the accession numbers MH458253-MH458269.

### Ecological niche model

**Figure 7:**
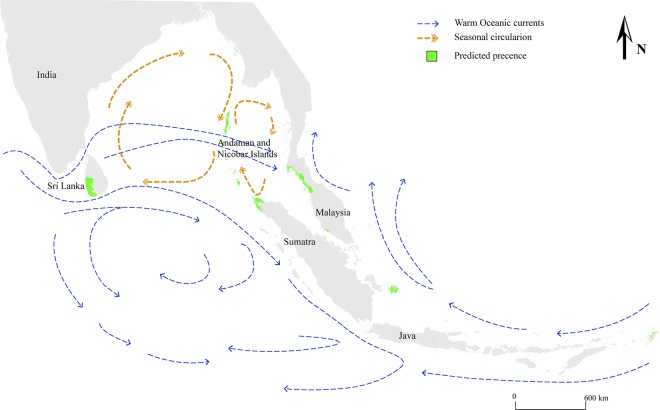
Predicted distribution of *C. zeylanica* derived from maximum entropy modeling. The oceanic currents related to Indian Ocean Gyre are represented in arrows.

We obtained the best AUC value of 0.975 for Receiver Operating Characteristic (ROC) Curve and the best Omission/Commission curves at the β regulation parameter of 5. The training omission rate was slightly higher than the test omission rate (ROC and AUC curves in Supplementary Fig. S1). Upon the total predicted distribution, Mean Diurnal Range had 53.9% of variable importance. Precipitation of the driest month and isothermality had 16.8% and 10.8% variable importance respectively. The predicted distribution derived from ENM shows Andaman and Nicobar Islands, Northern part of the Sumatra Island, Southern part of Thailand extending into northern part of Peninsular Malaysia as the predicted favourable niche for *C. zeylanica* complex *nov*. (Fig. 7). Apart from South East Asia, the model predicted a low level of distribution in Southern parts of the Western Ghats in India. The predicted distribution of *C. zeylanica* complex *nov*. is in line with our sampling locations as lowland Wet Zone of the country is the envisaged niche in Sri Lanka. This strengthens the accuracy and reliability of the model applied in the analysis.

## Discussion

Based on the morphological, molecular and climatic features of the studied taxa, the present study reveals that the Sri Lankan *Cycas* populations of the subsection *Rumphiae* mostly resemble *Cycas zeylanica*. In the *trnH-psbA* phylogeny (Fig. 6), *C. rumphii* diverged out from the clade containing the Sri Lankan subsection *Rumphiae* species.

We detected a high morphological variation in some vegetative traits as well as in some female reproductive features of *C. zeylanica*. This could be an artifact of hybridization among different congeneric species, and accordingly, we rename the studied taxa as *C. zeylanica* complex. The variation of many of the examined vegetative features appears to be so shallow and could not be used to distinguish different morphological groups (Supplementary Tables S4 and S5). In contrast, the female reproductive features considered in our study appear to be exhibiting significant variations. Of these, we identified five discriminating morphological traits that contribute to the variation of female reproductive structures of the *C. zeylanica* complex and these could be used in field identification of the species. The infertile region of the megasporophyll is the key feature in which all the significantly variable traits were detected (ratio of LIR/MWIR, ratio of LIR/LFR, LIR, NLS and MIR) (Supplementary Tables S4 and S5). Absence of apical crest-like structure in the seeds^8^ is also a feature that can be used in differentiating *C. zeylanica* from *C. rumphii*, but we were unable to use this feature as mature seeds were not available in all samples examined.

Our results further revealed that *C. zeylanica* complex in Sri Lanka can be assembled into six morphological groups with respect to female reproductive traits (Fig. 5). The Clusters B, D and F show independently unique features among them (MIR, ratio of LIR to MWIR and LIR). In contrast, the clusters A, C and E have some features derived from B, D and F clusters, indicating a possible mixing of *C. zeylanica* complex within Sri Lanka. Possibly, individuals belonging to Clusters B, D and F could be serving as the maternal populations, and all these findings support the theory of hybridization in *Cycas*^50–53^. A very low genetic divergence was observed in the phylogeny of the Sri Lankan *C. zeylanica* populations and this could be due to high gene flow among different morphotypes through hybridization. The possibility of hybridization among the congeneric members of subsection *Rumphia*e or their ancestors have also been observed by Keppel *et al*.^19^, and there is a possibility that these hybridized seeds may have dispersed over long distances aided by ocean currents or humans.

It appears that *C. zeylanica* complex in Sri Lanka is derived from multiple introductions from South East Asia. As depicted in the shape of the infertile region of the megasporophyll, one individual of the Cluster E (R12) (Fig. 5) resembles those of *Cycas pschannae* and *Cycas andamanica*. Semi-orbicular shape of the infertile region and presence of distinct hook-like structure in the margin of the base of the sterile portion were prominent features of the megasporophylls. Moreover, megasporophylls of Cluster D show a close resemblance to that of *C. edentata* (native range: Indonesia, Malaysia, Myanmar, Philippines, Singapore, Thailand and Vietnam), by the presence of lengthy infertile regions with entire margins. The individual R39 of Cluster A resembles *Cycas javana* (enigmatic taxa^54^ endemic to Java Islands) with respect to the arrangement of lateral spines and size of the infertile region, which is strongly supported by the ML tree where *Cycas edentata* and *C. javana* are clustered together with *C. zeylanica* complex (Fig. 6). Besides, individuals of the Cluster B show morphological similarities to *Cycas falcata*, which grows in Indonesia-Sulawesi Islands, by the presence of prominent lengthy lateral spines in the sterile region of the megasporophyll. In our phylogenetic tree, this is sister to the clade containing *C. zeylanica* complex (Fig. 6), confirming close evolutionary relationships. All these imply that convergence of species of the subsection *Rumphiae* may have taken place in Sri Lanka. This is in parallel with MacArthur and Levins’s^55^ finding that two similar species could produce a third intermediate species and ultimately will converge towards the nearer of the pair.

Although the vegetative morphological variations contributed less in forming above groups, PSP showed a continuous distribution among samples observed. Many individuals have PSP of 90 (Fig. 2A) indicating that they are frequently undergoing hybridization. Similarly, the very high variability and continuous nature of the distributions observed for LLL, LL, LL/LW, NL, LSP and CL also support the hypothesis of frequent mixing by hybridization. The observed traits could be controlled either by major genes or polygenes. However, the feature IAR showed a strict dimorphic nature and the observed ratio fits into modified epistatic ratio of 9:7 (i.e., complete gene-action). This could also be considered as important evidence for hybridization between extreme morphotypes. Similarly, the reproductive morphological feature, SLIR with the goodness of fit in modified epistatic ratio 15:1, strongly supports the hypothesis of hybridization events. These shreds of evidence propose multiple introductions of different South East Asian cycad species to Sri Lanka in the past and further hybridization within the island. As a result, a population with a unique genetic structure has been formed *in situ* (Fig. 6).

In Sri Lanka, previous studies have reported that individuals of the subsection *Rumphiae* have inherited a wide range of morphological diversity and had described these as forming a complex^8,50^. In light of these findings and in the absence of significant molecular and habitat-wise assessment, Mudannayake *et al*.^13^ have previously named Sri Lankan *Rumphiae* populations as *C. rumphii* complex. However, in agreement with Lindstrom and Hill^12^, the present study identified that the members of the Sri Lankan subsection *Rumphiae* are closer to *C. zeylanica* and the study group formed a well-supported unigeneric clade in the ML tree despite the high morphological diversity observed in female reproductive structures. Further, the niche of the study group overlaps with *C. zeylanica* niche^12^. Accordingly, we rename Sri Lankan *C. rumphii* complex as *C. zeylanica* complex.

The high morphological variation observed in female reproductive and some vegetative features of *C. zeylanica* complex had led to create taxonomic ambiguities not only in Sri Lanka^13^ but also elsewhere in Asia. According to available literature, several new species have been described in the recent past under the subsection *Rumphiae* from the Andaman Islands and sometimes from adjacent areas, but later, some of these have been reported as synonyms of previously named species or recognized to have many similarities to previously named species (Supplementary Table S8). For instance, *Cycas sainatii* has been reported as allied to *C. zeylanica*^56^, *Cycas darshii* allied to *C. rumphii*^57^, and *C. pschannae* allied to both *C. zeylanica* and *C. sainatii*^58^. Meanwhile, in 2015, another *Cycas* population was recorded from the coast of Middle and North Andaman Islands^59^. However, the species is considered as allied to both *C. zeylanica* and *C. edentata*. More recently, Sing^60^ has described *Cycas dharmarajii* which is also endemic to Andaman Islands. The species is reported as allied to both *C. zeylanica* and *C. pschannae*. However, Calonje *et al*.^2^ have stated that *C. andamanica* and *C. dharmarajii* are synonyms of *C. sainatii*. Such frequent ambiguities associated with cycads, especially under the subsection *Rumphiae* may be due to their high morphological variations, which may be an artifact of hybridization. Therefore, it is necessary to study these species at molecular level also to reveal evolutionary relationships of species in the subsection *Rumphiae* and the possibility of convergence of subsection *Rumphiae* species in other locations. As there are no *trnH-psbA* sequences available for the species *C. sainatii*, *C. dharmarajii*, *C. andamanica*, and *C. darshii*, and only a partial sequence of *trnH-psbA* is available for *C. pschannae* (GenBank accession MF977913) in GenBank database, we did not include these species in our phylogenetic analysis.

*Cycas zeylanica* is recorded only from Sri Lanka, Andaman and Nicobar Islands (evergreen littoral forests of Havelock Island, secondary littoral forest of West coast and beach vegetation of Kodiaghat in south Andaman)^12^. This distribution overlaps with our niche model analysis and it is more disposed to climatic restriction hypothesis. The niche of *C. zeylanica* in Sri Lanka overlaps with that in other native countries^12^. On the one hand, due to the favorable niche found in the lowland Wet Zone in Sri Lanka, it is possible for *C. zeylanica* to grow naturally in the area. On the other hand, it is also possible that *C. zeylanica* and other congeneric species of the subsection *Rumphiae* reached Sri Lanka through Long Distance Dispersal (LDD) by oceanic currents (Fig. 7) and colonized especially the coastal areas. Our proposal of multiple introductions of South East Asian cycads of the subsection *Rumphiae* forming *C. zeylanica* complex in Sri Lanka is confirmed by the ENM analysis too. The predicted distribution of this complex in South East Asia overlaps with the distributions of *C. sainatii, C. pschannae, C. dharmarajii, C. andamanica, C. darshii* and *C. edentata* and it is possible that these reached the island at different times.

Such LDD is presumed to affect the Sri Lankan flora. For instance, the diversity of some plant taxa of Sri Lanka is reported to have been originated by LDD from India, in the Paleozoic ice ages, through migratory animals^61^. Further, LDD has been reported for some other plant species such as mangroves (*Rhizophora apiculata* and *Rhizophora mucronata*^62^) and coconut (*Cocos nucifera* that grows in Sri Lanka^63^). However, this is the first record to support contribution of LDD in the floral diversification of cycads in Sri Lanka, and it is well known that cycads of the subsection *Rumphiae* undergo LDD due to the remarkable ability of its seeds to be buoyant in seawater^19^.

According to the current understanding, the dispersion of the subsection *Rumphiae* has occurred exclusively through LDD while South East Asia serves as the center of origin^17–19^. The warm oceanic currents and continuous seasonal circulations present in the Indian oceanic gyre could aid long distance gene flow frequently (Fig. 7). Besides, there is a possibility that seeds were brought to Sri Lanka from South East Asia by merchants during Portuguese and Dutch colonization periods (16^th^ to 17^th^ century) and planted them in different locations in the lowland wet zone. This may be the reason for having very old cycad plants in historical places such as old catholic churches in the southern Wet Zone of Sri Lanka. However, the establishment of congeneric species within the country could support continuous hybridization so as to maintain a low genetic divergence within *C. zeylanica* populations in the country.

Our entropy modeling exhibits climatic envelop structure of the population indicating that the *C. zeylanica* complex has undergone a major climatic isolation event. Individuals of the *C. zeylanica* complex are clearly restricted to the coastal areas with tropical humid climates. Being an island surrounded by the Indian Ocean, there is a chance for *C. zeylanica* complex to reach other coastal regions of the country but during field explorations, we observed that the species has not established in other climatic regions of the country.

In Sri Lanka, *C. zeylanica* is declared as a Critically Endangered species^64^ and the populations are also declining dramatically. Ramana *et al*.^65^ suggested that when considering the conservation of *C. zeylanica* the samples must be collected from diverse habitats of Sri Lanka. Lindstrom and Hill^12^ have reported that any viable regenerating populations of *C. zeylanica* in Sri Lanka is absent. In the current study, we have conducted an intense field survey spanning the Wet Zone of Sri Lanka and comprehended that no natural populations of the complex exist other than the hybridized and scattered individuals. A couple of Tsunami events had occurred around Sri Lanka in the past, causing severe damages to the coastal regions of the country, and as a result, the natural populations might have been adversely affected. During our field sampling, we noticed that local people deliberately cultivated the majority of these individuals, especially those found in the interior of the island. However, the population size is rather small, with probably less than 200 individuals existing in the entire country at present, highlighting the importance of strict conservation of the taxa.

## Conclusions

In the present study we renamed the Sri Lankan cycads of the subsection *Rumphiae* as *Cycas zeylanica* complex based on morphological and molecular sequence data. The higher morphological variation found in female reproductive structures distinguished the *C*. *zeylanica* complex *nov*. into six clusters; however, the genetic diversity among populations is very low. ENM suggests that the *C. zeylanica* complex prevails in South East Asia and the lowland Wet Zone of Sri Lanka. The morphological clustering suggests that long distance seed dispersal through sea currents and hybridization could have led to the formation *C. zeylanica* complex, and distribution within the region occurred due to human and animal interventions. The presence of a climatic envelop for the establishment of *C. zeylanica* complex in the country is evident. Due to the low population density and threats of extinction, *C. zeylanica* complex in Sri Lanka needs urgent conservation measures.

## Supplementary information

Supplimentary informations

## Data Availability

The DNA sequences generated during the current study are available in GenBank® under the accession numbers MH458253-MH458269 and the other datasets produced during and/or analyzed are available from the corresponding author on reasonable request.
