# Supplementary material for: The morpho-genetic and ecological niche analyses reveal the existence of climatically restricted Cycas zeylanica complex in Sri Lanka
Source: Sci Rep. 2019 Nov 14;9:16807. doi: 10.1038/s41598-019-53011-w (PMC6856184; doi:10.1038/s41598-019-53011-w)

The morpho-genetic and ecological niche analyses reveal the existence of climatically restricted *Cycas zeylanica* complex in Sri Lanka

Asanka Mudannayake[1,2], Lahiru Ranaweera[3], Preminda Samaraweera[2,3], Suneth Sooriyapathirana[2,3] and Anoma Perera[1,2*]

[1] Department of Botany, University of Peradeniya, Peradeniya, Sri Lanka.

[2] Postgraduate Institute of Science, University of Peradeniya, Peradeniya, Sri Lanka.

[3] Department of Molecular Biology and Biotechnology, University of Peradeniya, Peradeniya, Sri Lanka.

* Corresponding Author: anoma29@gmail.com

Table S1. Morphological features that considered during sampling.

	No.	Trait	Abbreviation	Description	Informative-ness
**Vegetative**	1	Percentage spines on the petiole	PSP	Length of the petiole with spines with respect to the total length of the petiole	P*
	2	Length of the petiole (cm)	LP	Length from base to the first leaf of the petiole	P, D
	3	Circumference of the petiole (cm)	CP	Petiole circumference at the position of the first leaflet	P*
	4	Length of the leaf lamina (cm)	LLL	Length from the first leaflet to the furthest leaflet	P*
	5	Full leaf length (cm)	FL	Total length of leaf lamina and the petiole	P, D
	6	Leaflet length (cm)	LL	Length from base to the tip of median leaflets	P*
	7	Leaflet width (cm)	LW	Width at the broadest position of a median leaflet	P*
	8	Ratio of leaflet length to width	LL/LW		P*
	9	Number of leaflets	NL	Total number of leaflets on bothside of the rachis	P*
	10	Ratio of leaflet number to length of the leaf lamina	NL/LLL		P*
	11	Leaflet spacing (cm)	LSP	Distance between midribs of adjacent median leaflets	P*
	12	Appearance of midrib above	MA	**Raised**, flat	M
	13	Appearance of midrib below	MB	Raised, **flat**	M
	14	Leaflet margin	LM	Entire	M
	15	Leaflet apex	LA	Accuminate	M
	16	Insertion angle to rachis (in degrees)	IAR	Insertion angle at the position of attachment of median leaflets to the rachis	P*
	17	Colourof mature leaflets	CML	Dark greeen	M
	18	Colour of young leaflets	CYL	**Light green**, bluish green	M
	19	Presence of tomentum on young leaflets	TYL	Present	M
	20	Colour of the tomentum	CT	Brown, **golden brown**	M
	21	Presence of cataphyll	PC	Persistent	M
	22	Cataphyll shape	CS	**Linear**, triangular	M
	23	Cataphyll length (cm)	CL	Length from base to the tip of the cataphyll	P*
	24	Length of the fertile region (cm)	LFR	Length from the position of first ovule to the last ovule	P*
	25	Length of the infertile region(cm)	LIR	Legth from the last ovule to the tip including the apical spine if present	P*
	26	Total length (cm)	TL	The total length of the fertile region and the infertile region	LV

**Megasporophyll**	27	Maximum width of the infertile region(cm)	MWIR	Width at the broadest position of the infertile region of megasporophyll	P*
	28	Ratio of the infertile region length to the maximum width	LIR/MWIR		P*
	29	Ratio of infertile region length to fertile region length	LIR/LFR		P*
	30	Margin of the infertile region	MIR	**Sharply dentate**, obscurely dentate, **slightly dentate towards distal end**, **entire to bumps**	P*
	31	Shape of the lamina of the infertile region	SLIR	Narrowly deltoid, eliptic to lanceolate, **linearly lanceolate to linear eliptical**, **orbicular-semi orbicular**	P*
	32	Number of lateral spines	NLS		P*
	33	Length of lateral spine (mm)	LLS	Length from base to the tip of the spine	LV
	34	Width of lateral spine(mm)	WLS	Width at the base of the spine	LV
	35	Length of the apical spine (cm)	LAS	Length from base to the tip	P*
	36	Number of ovules	NO	Both young and mature structures were considered and total number of fertilized and aborted ovules were considered in mature structures	P*
	37	Length of the seed (cm)	LS		M
	38	Width of the seed (cm)	WS		M
	39	Ratio of seed length to width	LS/WS		M
	40	Seed colour	SC	**Orange brown**, yellow	M
	41	Seed shape	SS	**Ovoid**, subglobose	M
	42	Presence of fibrous layer in the sarcotesta of the seed	PFS	Present, **absent**	M
	43	Presence of spongy endocarp layer of the seed	PSS	**Present**, absent	M
**Microsporophyll**	44	Length of the fertile region (cm)	LFRM	Length of the fertile region where microsporangia are present	NC
	45	Maximum width (cm)	MW	Width at the broadest position of the fertile region	NC
	46	Length of the apical spine(cm)	LAS	Length from base to the tip of the apical spine	NC
	47	Arrangement of apical spine	AAS	Curved, upright	NC
	48	Shape of the pollen cone	SPC	Narrowly ovoid, ovoid-oblong	NC
	49	Pollen cone colour	PCC	Pale fawn, brown	NC

P-polymorphic, D-dependant traits, LV-Less variable, M-monomorphic, NC-not considered, * traits used in the analysis
categorical characters in bold reprents by the subsection *Rumphiae*

Source: Mudannayake A, Sooriyapathirana S, Samaraweera P, Perera A (2015) *Cycas* Taxa in Sri Lanka and their morphological characteristics of taxonomic significance.
Ceylon Journal of Science (Biological Sciences) 44. doi:10.4038/cjsbs.v44i1.7337.

Table S2. Metadata of the t*rnH-psbA* sequences used in the phylogenetic analysis.

Morphological Grouping		Taxa	Marker Length (bp)	Accession No.	Reference
**Section *Indosinenses* J.Schust.**		*Cycas lindstromii* S.L. Yang, K.D. Hill and Hiäp	509	KX182268	Liu et al. 2018
		C. clivicola K.D. Hill	481	KX182245	Liu et al. 2018
**Section *Panzhihuenses* (D.Yue Wang) K.D.Hill**		*C. panzhihuaensis* L.Zhou & S.Y.Yang	508	KP117157	Wei et al. 2015
**Section *Asiorientales* J. Schust.**		*C. revoluta* Thunb.	162489	JN867588	Liu et al. 2018
		C. taitungensis C.F.Shen, K.D.Hill, C.H.Tsou & C.J.Chen	487	KX182318	Liu et al. 2018
**Section *Stangerioides* Smitinand**		*C. guizhouensis* Lan & R.F.Zou	482	KX182258	Liu et al. 2018
		C. bifida (Dyer) K.D.Hill	481	KX182229	Liu et al. 2018
**Section *Wadeae* K.D.Hill & A.Lindstrom.**		*C. wadei* Merrill	455	KX182325	Liu et al. 2018
		C. curranii (J.Schust.) K.D.Hill	455	KX182239	Liu et al. 2018
	**Subsection *Endemicae* Schuster**	*C. silvestris* K.D. Hill	491	KP117161	Wei et al. 2015
		C. media sub sp banksii K.D. Hill	495	KX182276	Liu et al. 2018
		C. campestris K.D. Hill	485	KX182241	Liu et al. 2018
	**Subsection *Cycas* Greuter**	*C. indica* A.Lindstrom & K.D.Hill	481	KX182263	Liu et al. 2018
		C. spherica Roxb.	481	KX182312	Liu et al. 2018
		C. beddomei Dyer	481	KX182230	Liu et al. 2018
		C. circinalis L.	481	KX182242	Liu et al. 2018
		C. circinalis L.	581	MF348799	Zúñiga et al. 2017
		C. javana (Miq.) de Laub	485	KX182265	Liu et al. 2018
		C. nathorstii J. Schust	486	KX182289	Liu et al. 2018
		C. montana A.Lindstr. & K.D.Hill	480	KX182288	Liu et al. 2018
		C. lacrimans A.Lindstr. & K.D. Hill	485	KX182266	Liu et al. 2018
		C. aenigma K.D.Hill & A.Lindstrom	476	KX182220	Liu et al. 2018
		C. bougainvilleana K.D. Hill	476	KX182228	Liu et al. 2018
		C. zeylanica (J. Schust.) A. Lindstrom & K.D. Hill	491	KX182329	Liu et al. 2018
		C. apoa K.D.Hill	485	KX182223	Liu et al. 2018

**Section *Cycas (Lemuricae* Schuster*)***		*C. inermis* Lour.	481	KX182264	Liu et al. 2018
		C. rumphii Miq.	476	KX182303	Liu et al. 2018
		C. scratchleyana F.Muell	489	KX182313	Liu et al. 2018
		C. falcata K.D.Hill	485	KX182252	Liu et al. 2018
		C. edentata de Laub	485	KX182249	Liu et al. 2018
		Cycas zeylanica complex	567		Present study
		Cycas zeylanica complex	567		Present study
		Cycas zeylanica complex	567		Present study
	**Subsection *Rumphiae* K. D. Hill**	*Cycas zeylanica complex*	567		Present study
		Cycas zeylanica complex	567		Present study
		Cycas zeylanica complex	567		Present study
		Cycas zeylanica complex	567		Present study
		Cycas zeylanica complex	567		Present study
		Cycas zeylanica complex	567		Present study
		Cycas zeylanica complex	567		Present study
		Cycas zeylanica complex	567		Present study
		Cycas zeylanica complex	567		Present study
		Cycas zeylanica complex	567		Present study
		Cycas zeylanica complex	567		Present study
		Cycas zeylanica complex	567		Present study
		Cycas zeylanica complex	567		Present study
		Cycas zeylanica complex	567		Present study

Liu, J., Zhou, W. & Gong, X. Species delimitation, genetic diversity and population historical dynamics of Cycas diannanensis (Cycadaceae) occurring sympatrically in the Red River region of China. Front Plant Sci. 6, 696. doi:10.3389/fpls.2015.00696 (2015).

Wei Z, Meng-Meng G, Xun G (2015) Cycas chenii (Cycadaceae), a new species from China, and its phylogenetic position. Journal of systematics and evolution, 53:489-498.

Zúñiga, J.D. et al. Data Release: DNA barcodes of plant species collected for the Global Genome Initiative for Gardens Program, National Museum of Natural History, Smithsonian Institution. PhytoKeys. 88, 119. doi: 10.3897/phytokeys.88.14607 (2017).

Table S3. Bioclimatic variables and its variable importance in Maximum Entropy Modeling.

Bioclimatic Varible	Percentage contribution (%)
Annual Mean Temperature	53.9
Mean Diurnal Range [Mean of monthly (max temp - min temp)]	16.8
Isothermality	10.8
Temperature Seasonality	5.5
Max Temperature of Warmest Month	4.0
Min Temperature of Coldest Month	3.3
Temperature Annual Range	2.0
Mean Temperature of Wettest Quarter	2.0
Mean Temperature of Driest Quarter	1.9
Mean Temperature of Warmest Quarter	0.0
Mean Temperature of Coldest Quarter	0.0
Annual Precipitation	0.0
Precipitation of Wettest Month	0.0
Precipitation of Driest Month	0.0
Precipitation Seasonality [Coefficient of Variation]	0.0
Precipitation of Wettest Quarter	0.0
Precipitation of Driest Quarter	0.0
Precipitation of Warmest Quarter	0.0
Precipitation of Coldest Quarter	0.0

Table S4. Pearson correlation coefficients for quantitative traits of vegetative and female reproductive morphological features.

	PSP	CP	LLL	LL	LW	LL/LW	NL	NL/LLL	LSP	CL	MWIR	NO	LAS	LFR	LIR	LIR/MWIR
CP	-0.31															
LLL	0.30	-0.19														
LL	-0.08	0.07	0.16													
LW	0.08	0.27	-0.11	-0.18												
LL/LW	-0.21	-0.15	0.02	0.61***	-0.83***											
NL	0.09	-0.25	0.71***	0.04	-0.12	0.01										
NL/LLL	-0.25	-0.10	-0.35	-0.14	-0.03	0.01	0.40*									
LSP	0.36	0.12	0.04	-0.19	-0.15	-0.04	-0.14	-0.23								
CL	-0.04	0.29	-0.42*	-0.11	-0.18	0.10	-0.40*	0.05	0.26							
MWIR	0.06	0.05	0.06	-0.23	0.24	-0.35	0.10	0.06	0.00	-0.29						
NO	-0.05	0.15	0.23	0.10	-0.26	0.18	0.23	0.01	0.06	0.10	0.27					
LAS	0.29	0.17	-0.03	0.28	-0.12	0.20	0.09	0.16	0.29	0.06	0.23	0.59***				
LFR	-0.34	0.16	0.09	0.13	-0.17	0.12	0.25	0.18	-0.08	-0.33	0.32	0.53**	0.27			
LIR	-0.10	0.33	-0.24	0.07	-0.19	0.16	-0.20	0.05	0.28	0.39*	0.05	0.09	0.40*	-0.30		
LIR/MWIR	-0.11	0.28	-0.26	0.20	-0.31	0.35	-0.24	0.02	0.24	0.50**	-0.50**	-0.04	0.27	-0.41*	0.83***	
LIR/LFR	0.25	0.10	0.13	0.00	0.01	0.03	-0.21	-0.08	0.21	0.43*	-0.14	-0.23	0.15	-0.83***	0.74***	0.71***

PSP: % spines on the petiole; CP: circumference of the petiole (cm); LLL: length of the leaf lamina (cm); LL: leaflet length (cm); LW: leaflet width (cm); LL/LW: ratio of LL to LW; NL: no. of leaflets; NL/LLL: ratio of NL to LLL; LSP: leaflet spacing (cm); CL: cataphyll length (cm); MWIR: maximum width of the infertile region (cm); NO: no. of ovules; LAS: length of the apical spine (cm); LFR: length of the fertile region (cm); LIR: length of the infertile region (cm); LIR/MWIR: ratio of LIR to MWIR; LIR/LFR: ratio of LIR to LFR

Note: *$p \leq 0.05$, ** $p \leq 0.01$, *** $p \leq 0.001$

Table S5. Spearman rank correlation coefficients for qualitative traits of vegetative and female reproductive morphological features.

	MIR	SLIR	NLS
SLIR	0.18		
NLS	-0.67***	-0.08	
IAR	0.35	0.25	-0.35

MIR: margin of the infertile region; SLIR: shape of the lamina of infertile region; NLS: no. of lateral spines; IAR: insertion angle to rachis

Note: *$p \leq 0.05$, ** $p \leq 0.01$, *** $p \leq 0.001$

Table S6. Eigen values, percentage variation and cumulative variation for the calculated principal components.

PC	Proportion of the varience	Cumulative varience	Eigen value
1	16.171	16.171	4.366
2	14.476	30.647	3.908
3	11.225	41.872	3.031
4	8.683	50.555	2.344
5	7.925	58.480	2.140
6	7.039	65.518	1.900
7	6.258	71.777	1.690
8	4.855	76.631	1.311
9	4.564	81.195	1.232
10	3.728	84.923	1.007
11	3.104	88.027	0.838
12	2.768	90.795	0.747
13	2.505	93.301	0.676
14	2.070	95.371	0.559
15	1.422	96.793	0.384
16	1.031	97.824	0.278
17	0.885	98.709	0.239
18	0.519	99.228	0.140
19	0.378	99.606	0.102
20	0.187	99.793	0.051
21	0.120	99.913	0.032
22	0.066	99.979	0.018
23	0.018	99.997	0.005
24	0.003	100.000	0.001

Table S7. Variable sites identified in *trnH-psbA* gene region of the species considered in phylogenetic analysis (SNP- Single Nucleotide Polymorphism; INDEL- Insertion/delition; Pi- parsimony informative sites; S-Singleton sites)

SNP or INDEL position	Pi	S	No. of allels	type of allels
19	×	√	3	C/T/-
54	×	√	3	G/T/-
65	×	√	2	C/T
101	√	×	3	A/T/-
102	√	×	3	A/T/-
131	√	×	3	A/T/-
132	√	×	3	A/T/-
134	√	×	3	A/T/-
135	√	×	3	A/T/-
151	×	√	2	C/T
154	×	√	2	C/T
155	×	√	2	A/T
162	√	×	3	A/T/-
186	×	√	2	C/T
191	√	×	2	C/T
249	×	×	3	A/T/-
257	×	√	2	A/G
270	×	√	3	A/T/-
271	×	√	3	A/T/-
272	×	√	3	G/T/-
274	×	√	3	G/A/-
279	√	×	3	C/T/-
311	√	×	2	A/G
323	√	×	2	C/A
326	√	×	2	G/T
450	√	×	2	C/T
483	√	×	2	A/C
503	√	×	2	A/G
529	√	×	2	G/T
585	×	√	2	G/T
586	×	√	2	C/G

Table S8. Possible taxonomic ambiguities in recently named *Cycas* species of the subsection *Rumphiae*.

New species	Natural distribution	Reported taxonomic ambiguities
Cycas sainatii R.C. Srivastava	Andaman and Nicobar Islands (Srivastava 2014a)	allied to *C. zeylanica* [56]
Cycas darshii R.C.Srivast. & B. Jana	Andaman and Nicobar Islands (Srivastava 2014b)	allied to *C. rumphii* [57]
Cycas pschannae R.C.Srivast. & L.J.Singh	Andaman and Nicobar Islands (Srivastava and Sing 2015)	allied to both *C. zeylanica* and *C. sainatii* [58]
Cycas andamanica K. Prasad, M. V. Ramana, Sanjappa & B.R. P. Rao	along the coast of Middle and North Andaman Islands; endemic to Andaman islands (Prasad et al. 2015)	allied to both *C. zeylanica* and *C. edentata* [59] synonym of *C. sainatii* [2]
Cycas dharmarajii L.J. Sing	Andaman Islands (Sing 2017)	allied to both *C. zeylanica* and *C. pschannae* [60] synonym of *C. sainatii* [2]

Figure S1. Receiver Operating Characteristic (ROC) Curve and Omission/ Commission curve derived from Ecological Niche Modeling.

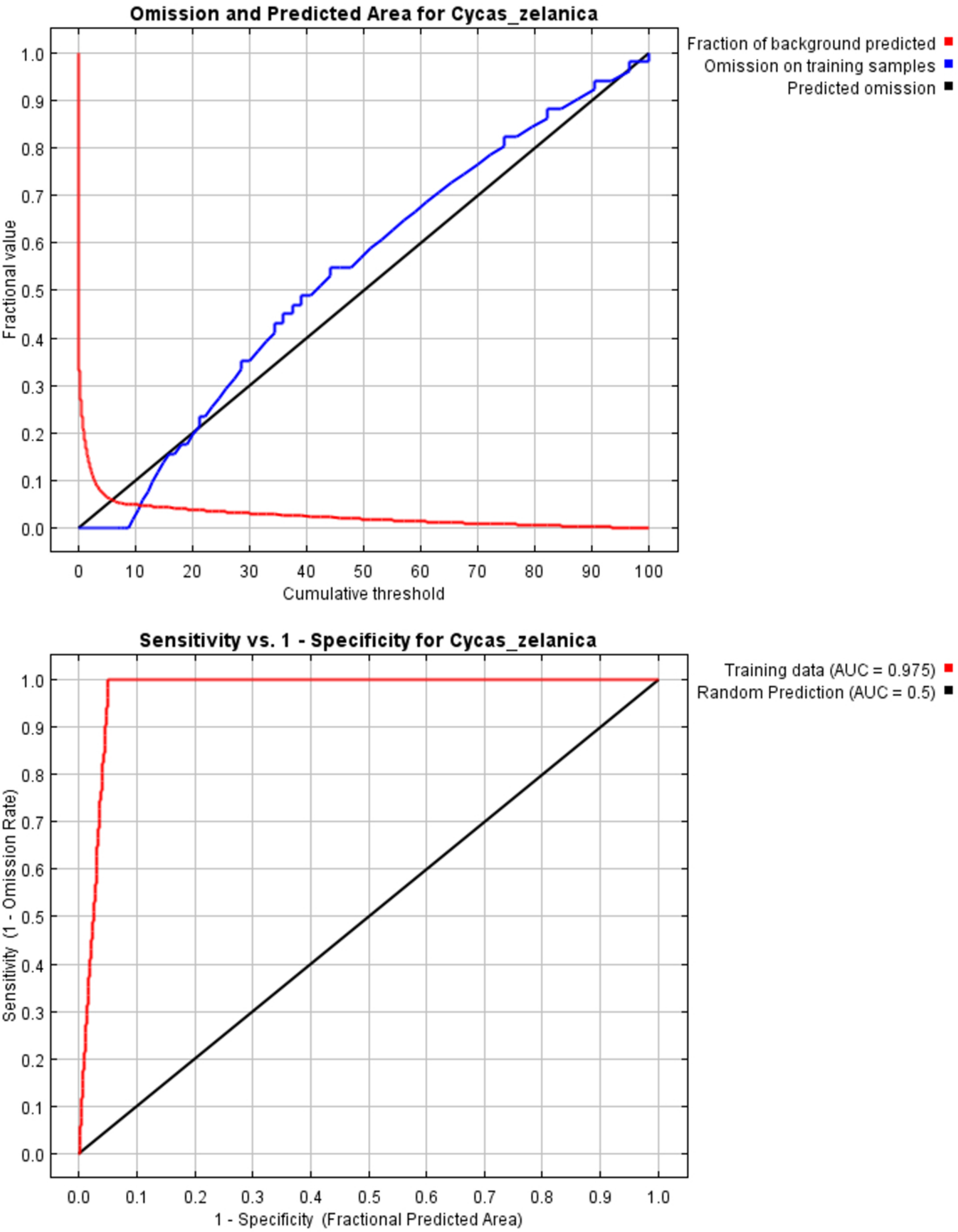

Supplement: Supplementary file 1 — Supplimentary informations [file 41598_2019_53011_MOESM1_ESM.pdf]
